# A Randomized, Placebo-Controlled Trial Evaluating Safety and Immunogenicity of the Killed, Bivalent, Whole-Cell Oral Cholera Vaccine in Ethiopia

**DOI:** 10.4269/ajtmh.14-0683

**Published:** 2015-09-02

**Authors:** Sachin N. Desai, Zenebe Akalu, Samuel Teshome, Mekonnen Teferi, Lawrence Yamuah, Deok Ryun Kim, Jae Seung Yang, Jemal Hussein, Ju Yeong Park, Mi Seon Jang, Chalachew Mesganaw, Hawult Taye, Demissew Beyene, Ahmed Bedru, Ajit Pal Singh, Thomas F. Wierzba, Abraham Aseffa

**Affiliations:** International Vaccine Institute, Seoul, Korea; Armauer Hansen Research Institute, Addis Ababa, Ethiopia

## Abstract

Killed whole-cell oral cholera vaccine (OCV) has been a key component of a comprehensive package including water and sanitation measures for recent cholera epidemics. The vaccine, given in a two-dose regimen, has been evaluated in a large number of human volunteers in India, Vietnam, and Bangladesh, where it has demonstrated safety, immunogenicity, and clinical efficacy. We conducted a double-blind randomized placebo-controlled trial in Ethiopia, where we evaluated the safety and immunogenicity of the vaccine in 216 healthy adults and children. OCV was found to be safe and elicited a robust immunological response against *Vibrio cholerae* O1, with 81% adults and 77% children demonstrating seroconversion 14 days after the second dose of vaccine. This is the first study to evaluate safety and immunogenicity of the vaccine in a population outside Asia using a placebo-controlled, double-blind, randomized study design.

## Background

Cholera remains a serious global challenge with disease burden estimated at over 3 million cases and more than 100,000 deaths throughout much of the developing world.^1^ Many large and prolonged outbreaks have resulted in cholera becoming endemic not only in many parts of Africa, but also in Haiti, where cholera had not been previously reported for nearly a century.^2^ In recent years, the World Health Assembly has called for the incorporation of oral cholera vaccines (OCVs) as part of an integrated, comprehensive strategy of cholera prevention and control.^3^ Currently, two killed OCVs are prequalified by the World Health Organization (WHO). Both Dukoral (Crucell, Leiden, The Netherlands), a whole-cell vaccine supplemented with recombinant cholera toxin B subunit (WC-rBS), and Shanchol (Shantha Biotechnics Ltd., Hyderabad, India), a bivalent whole-cell vaccine without cholera toxin (WC), have demonstrated immunogenicity^4^–^6^ and efficacy^7^,^8^ in preventing cholera in endemic settings. Because Shanchol is less expensive and does not require the coadministration of buffer, it can be delivered in a faster and less cumbersome manner. This has been corroborated during recent preventive and reactive campaigns in Bangladesh, India, Haiti, South Sudan, and Guinea.^9^–^12^

In Ethiopia, “acute watery diarrhea” (AWD) and cholera have been used interchangeably, largely due to limited diagnostic capacity. Since 1993, yearly AWD outbreaks, encompassing various infectious etiologies including laboratory-confirmed cholera, have affected numerous regions throughout sub-Saharan Africa.^13^ Severe diarrhea caused by *Vibrio cholerae* O1 has been an important cause of morbidity and mortality in Ethiopia, with the Federal Ministry of Health reporting over 22,000 cases and 219 deaths (case fatality rate 1%) in 2006.^14^ AWD due to *V. cholerae* O1 has been reported in five regions, with the most populous region (Oromia) reporting cholera in five out of six years during the 2006–2011 period.^15^ Cholera outbreaks in developing countries occur in both endemic and epidemic settings. Populations with endemic cholera presumably have high levels of preexisting immunity following age-related acquisition patterns because of recurrent cholera exposures. Conversely, outbreaks in less endemic settings tend to occur in populations with limited preexisting immunity. This helps to explain why younger children with less background immunity have make up a higher proportion of cases in endemic settings, while cholera incidence tends to be age independent in epidemic settings. The killed bivalent formulation of OCV has been evaluated in a large number of human volunteers in Vietnam, India, and Bangladesh, where its safety, immunogenicity,^5^,^6^,^16^ and clinical protective efficacy have been demonstrated.^8^ During a recent OCV campaign in rural Haiti, investigators assessed immune response in a region without recent historical exposure to *V. cholerae* and found the two-dose OCV regimen to be highly immunogenic in Haitian adults and children.^17^ In this placebo-controlled, double-blind randomized trial, we evaluated the safety and immunogenicity of killed bivalent whole-cell OCV in a high-risk population residing in a less endemic region outside Asia.

## Methods

### Participants.

The study was conducted at the Clinical Trials Center of the Armauer Hansen Research Institute (AHRI) in an urban community near Addis Ababa, Ethiopia. Healthy adults (18 years and above) and children (aged 1–17 years) were recruited. Main exclusion criteria considered were pregnancy (as determined by urine pregnancy test for females aged 12 years and above), ongoing illness, immune-compromising conditions, diarrhea (defined as having three or more loose/watery stools within a 24-hour period during the past 6 weeks) and gastrointestinal symptoms (abdominal pain, loss of appetite, nausea, and vomiting) in the past 24 hours, or previous receipt of OCV. Written informed consent was obtained by study physicians for all adults and parents/guardians of participating children, as well as written assent for 12- to 17-year-old-participants.

### Regulatory and ethics approvals.

The trial protocol was approved by the Institutional Review Board of the International Vaccine Institute (IVI) in Seoul, Korea, the AHRI/All Africa Leprosy, Tuberculosis and Rehabilitation Training Center (ALERT) Ethics Review Committee (AAERC), the National Health Research Ethics Review Committee (NHRERC), and the Food, Medicine, and Healthcare Administration and Control Authority of Ethiopia (FMHACA) in Addis Ababa. The study was monitored by an independent local study monitor and external clinical monitors from IVI and Shantha Biotechnics Ltd (Hyderabad, India). Furthermore, an impartial Data and Safety Monitoring Board reviewed all the safety data from the trial. The trial was registered in ClinicalTrials.gov (NCT01524640).

### Study design.

This is an individually randomized, double-blind, placebo-controlled trial among healthy adults (aged 18 years and above) and children (aged 1–17 years) randomized into two groups at 1:1 ratio to receive two oral doses of either killed bivalent (O1 and O139) whole-cell OCV or a nonbiological placebo. Assuming a 5% diarrheal rate among placebo and vaccine recipients alike, to exclude a vaccine–placebo difference in the rate of diarrhea of greater than 20% (upper bound of the one-tailed 95% confidence intervals [CI]) with a power of 0.8, a minimum of 33 participants would be required in each intervention group. For serum vibriocidal responses, assuming a background rate of 5% seroconversion among placebo recipients after the second dose and a true rate of vibriocidal response in the vaccine group of 60% to exclude a vaccine–placebo difference of 30% with a power of 0.8, a minimum of 43 participants would be necessary for each group. Using the Farrington and Manning method of precision-based sample size calculation^18^ and adjusting for a 20% attrition rate, we calculated the need for 54 participants each in the vaccine and placebo groups, requiring a total of 108 adults and 108 children.

### Randomization and blinding.

Four lists of individualized numbers were computer generated by a statistician from IVI, who was not involved in the study, to achieve stratified randomization by age group: 1–5, 6–10, 11–17, and ≥ 18 years. Randomization was performed in variable blocks of 4–8, to ensure that both balance between intervention groups and blinding are maintained. Study agents were pre-labeled by Shantha Biotechnics personnel, who were not involved in the conduct or monitoring of the trial. The IVI, local safety monitor, and the manufacturer-held sealed copies of the randomization list, which was not opened until all data for analysis had been frozen and a hard copy had been given to the local monitor. All study personnel and participants were blinded to treatment assignment during the duration of the study.

### Intervention and administration.

Each dose of vaccine contained heat and formalin-killed whole-cell bacteria consisting of five strains of *V. cholerae* O1 and O139. The placebo consisted of a sugar-buffer solution, similar in appearance to the vaccine. Participants were randomized to receive two doses of vaccine or placebo at a 14-day interval and instructed not to eat 1 hour before and 30 minutes after dosing. After proper shaking and inspection of the vial by the study physician, a member of the field team administered the 1.5-mL dose of study agent, followed by offering the participant a small cup of water. All participants were observed for 30 minutes following dosing and were interviewed by research staff for three consecutive days after each dose to monitor for adverse events. Active surveillance via questioning at ensuing study visits was conducted for any adverse or serious adverse events for 28 days following the dosing regimen. All participants were instructed to contact study staff or visit the clinical trial study physician if any adverse events presented during the study period. Venous blood samples were obtained immediately before dosing on day 0, before dosing on day 14, and on day 28 (2 weeks following second dose). Serum samples were stored at −20°C and shipped to IVI, where vibriocidal antibody assays were performed.

### Outcomes.

We investigated whether the killed bivalent OCV was safe and immunogenic among adults and children residing in a less endemic area in Ethiopia. The primary endpoint for safety was the proportion of participants experiencing adverse events 3 days after dosing or severe adverse events within 14 days following either dose. For immunogenicity, the primary endpoint was to measure the proportion of participants demonstrating seroconversion, defined as a 4-fold or greater rise in titers of serum vibriocidal antibodies relative to baseline and 14 days after the second dose.

### Statistical and laboratory methods.

#### Statistical analysis.

Data were entered in Visual FoxPro 9.0 (Microsoft Corp., Redmond, WA) and analyses were performed in SAS 9.3 (SAS Institute, Cary, NC). Analyses for comparisons of dichotomous outcomes such as seroconversion were performed with the χ^2^ test or Fisher's exact test when an expected cell count was less than five. For comparisons of vibriocidal titers, Student's *t* test was performed using pooled method or Satterthwaite method depending on whether the variances were equal or not. Vibriocidal titers and fold increases were logarithmically transformed before statistical analyses. Nonparametric Wilcoxon rank-sum test and Kolmogorov–Smirnov test were performed depending on equality of variance if data were not normally distributed. Multiple linear regression models were fitted to assess vaccine effect after controlling confounding variable. In the model, the logarithms of vibriocidal titers at 2 weeks following the second dose was the dependent variable, and the vaccine status (vaccine or placebo), logarithm of the baseline vibriocidal titers, and confounding variable were fitted as the independent variables. The primary outcomes that were proportion of participants with adverse events within 3 days following dosing and vibriocidal seroconversion were evaluated with one-tailed 97.5% CI using the Wilson Score method.^19^ Statistical evaluations of all other comparisons were two tailed.

#### Laboratory analysis.

Venipuncture was performed before the first dose, 14 days after dose 1, and 14 days after dose 2 for all the 216 study participants enrolled in the study. At each study visit, 3 mL blood was collected from all participants. Serum was separated and stored in a dedicated freezer at −20°C in the microbiology laboratory at AHRI (Addis Ababa) and then shipped to IVI (Seoul) for analysis. The serum vibriocidal antibody titers were determined using the microtiter technique as previously described.^20^ An increase of titer by 4-fold or greater between baseline and postimmunization sera was considered to meet the criteria for seroconversion, which was a primary end point for the study. Vibriocidal titers were measured against O1 Inaba, O1 Ogawa, and O139.

## Results

### Enrollment.

**Figure 1.**
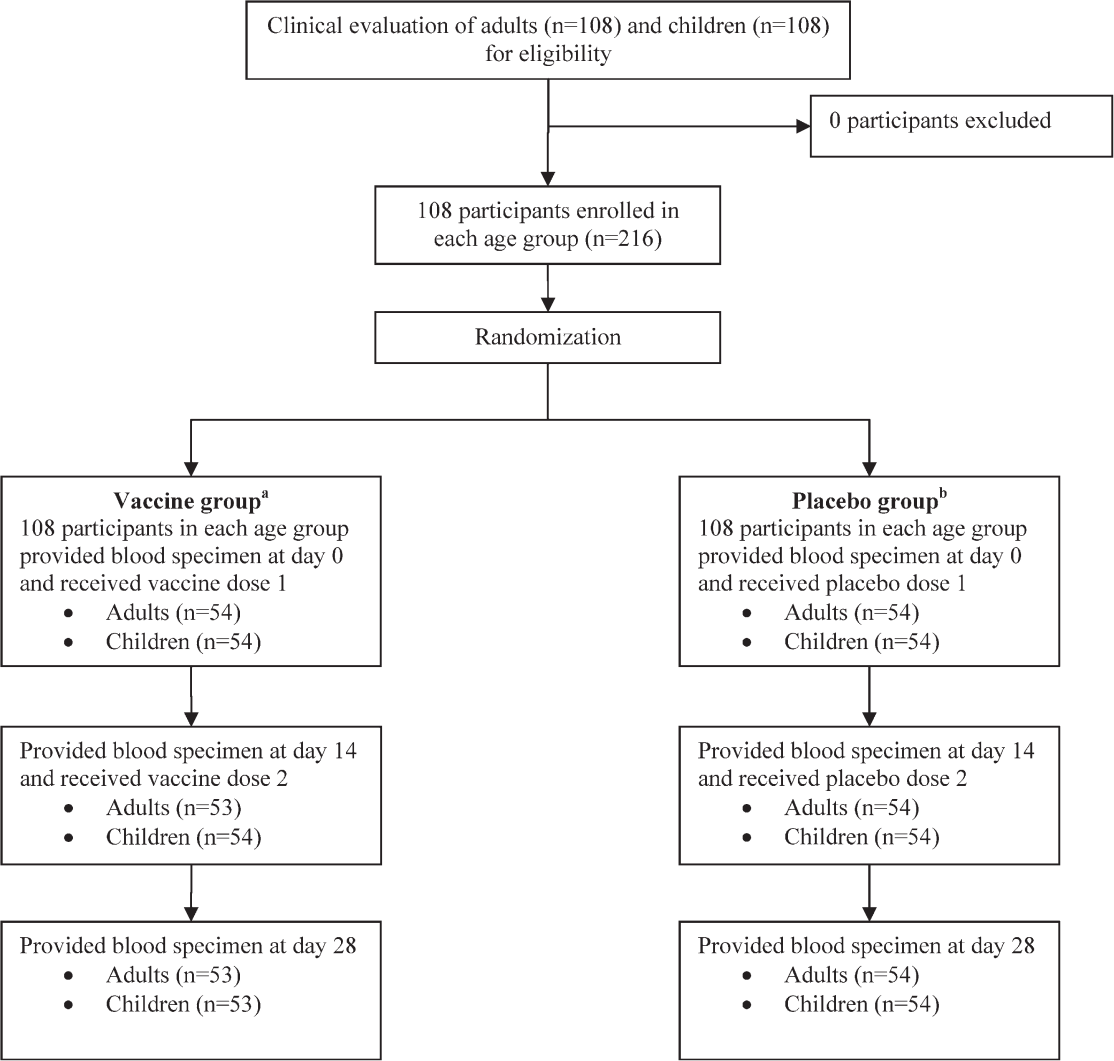
Enrollment of participants. ^a^One adult lost to follow up on day 14 and one child did not provide blood at day 28. ^b^One adult provided blood specimen 2 days earlier at day 28 and one child received partial dose 2.

Enrollment and follow-up of all participants are shown in Figure 1

. A total of 216 participants (108 adults, 108 children) were recruited from December 2012 to July 2013. Of the 216 participants, 106/108 adults and 106/108 children received both doses of the assigned study agent and provided all three blood samples. Characteristics of study participants are shown in Table 1. Age and weight were comparable between adult and child participants receiving either study agent. We had a significantly higher number of adult males receiving vaccine (59% versus 41%, *P* = 0.03), for which vaccine effect was adjusted using a multiple regression model.

### Safety.

All participants were included in the safety analysis. No significant differences in rates of adverse events were observed between the vaccine and placebo groups (Table 2). No adverse events were reported in any vaccine recipients within 3 days of dosing. Only one child who received vaccine reported of mild fever and sore throat within 14 days of dosing (day 12) and resolved following medication. No serious adverse events were reported during the trial.

### Immunogenicity: vibriocidal antibody titers.

A per protocol analysis was conducted for immunogenicity data, including 212 participants who completed all study visits. Table 3 illustrates baseline geometric mean titers (GMT), geometric mean fold rise (GMFr), and seroconversion rates as defined by a ≥ 4-fold rise in titers from baseline GMT to 14 days following the first and second doses. Among vaccine recipients, the baseline GMT to *V. cholerae* O1 Inaba was 3.5, 8.3, and 16.9 for 1- to 5-year-, 6- to 17-year, and ≥ 18-year age groups, respectively. GMFr of *V. cholerae* O1 Inaba vibriocidal antibody titers among vaccine recipients were 4.3 (1–5 years), 32.6 (6–17 years), and 15.1 (≥ 18 years) and demonstrated statistically significant differences compared with the placebo arms in both adults and children. Seroconversion rates against *V. cholerae* O1 Inaba was 89% in older children (6–17 years) and 81% in adults (≥ 18 years). Although children aged 1–17 years demonstrated a 77% seroconversion rate, responses were markedly lower (53%) in the youngest age group (1–5 years) (Table 4). Because of significant difference in enrollment of vaccinated male and female adults (*P* = 0.03, Table 1), a multiple regression model was used to adjust for sex. The crude and adjusted estimates of GMFr were similar and remained statistically significant (*P* < 0.001, Table 3) after correction for the higher number of vaccinated adult males. Baseline GMTs were low in both adults (16.9) and children (6.0), with significantly higher GMFr and seroconversion noted in the vaccine group. Overall, 23% adults and 18% children had baseline GMTs over 160.

Seroconversion rates against O1 Ogawa were significantly higher in vaccine group in 1–5, 6–17, and ≥ 18 years (75%, 90%, and 70%, respectively) compared with placebo recipients in the same age groups (0%, 6%, and 13%, respectively). Seroconversion rates against *V. cholerae* O1 Inaba were robust following first and second doses in adults (70%, 81%), as well as children (74%, 77%). Seroconversion against O1 Ogawa after each dose was also high in both adults (65%, 70%) and children (80%, 84%). Although a small sample size, children in the youngest age group (1–5 years) demonstrated higher seroconversion against O1 Inaba after a single dose (65%) compared with both doses (53%). No difference in seroconversion rates between the first and second doses was noted against O1 Ogawa in this age group (75% after each dose). Although the differences in GMFr and seroconversion rates for *V. cholerae* O139 were statistically significant in the vaccinated group compared with placebo recipients in all age groups, the values were much less pronounced compared with *V. cholerae* O1 Inaba and O1 Ogawa (Tables 3 and 4).

## Discussion

Adding to the recent work by Charles and others,^17^ these results support the claim that the killed bivalent OCV, Shanchol, can elicit a strong immune response in areas that are less endemic to *V. cholerae*. This bridging study is the first to evaluate safety and immunogenicity of this vaccine in a population outside Asia using a placebo-controlled, double-blind, randomized study design. Vibriocidal antibody response remains the most studied immunologic marker of cholera infection, with 40–80% individuals living in endemic areas having detectable antibodies by 15 years of age.^21^ Though this vaccine was found to be immunogenic in adults and children, the youngest age group demonstrated a lower response of GMFr and seroconversion. An earlier study in the Son La province of Vietnam,^16^ where cholera is less endemic, found elevated levels of GMFr in serum antibodies in adults (26.8), which was substantially increased when compared with similar populations in Kolkata (4.5),^5^ India, and Bangladesh (9).^6^ These observations, together with the findings from this study support the explanation that responses to the vaccine are inversely related to baseline serum vibriocidal antibody titer. Although higher titers have been associated with protection against disease,^22^ correlation with protection is incomplete.^23^ The absence of an age-related lowering of antibody response in this study is probably due to limited exposure to *V. cholerae* and a lack of preexisting antibody in older individuals. Hence, in less endemic regions, such as our study area, all ages respond as an immunologically naïve cohort. Understandably, this data differ from regions with continual exposure to cholera, such as India and Bangladesh, because of the amount of background natural cholera exposure. Still, a limitation of this trial is that it cannot be directly compared with other immunogenicity trials because of differences in study protocol and absence of standardization of vibriocidal titers across laboratories.

Intriguingly, the immunogenicity results of this OCV in Haitian vaccines revealed vibriocidal titers similar to age-matched cohorts from Bangladesh following a two-dose OCV regimen. Differences between the Haiti trial and the current Ethiopia trial may be explained by the fact that participants in Haiti may have developed background immunity since vibriocidal titers were measured in the middle of 2013, with the epidemic starting October 2010. This may represent an immunologically primed population by boosting underlying immunity. In endemic settings, children have been shown to be able to mount vibriocidal and toxin-specific antibody, as well as memory B cell responses comparable to that of adults.^24^ However, a diminished T cell response, as well as other important host factors in young children such as helminth coinfection, enteropathy, and micronutrient deficiencies^25^ can serve as contributing factors to explain lower observed immune responses in this age group. An important similarity between the Haitian and Ethiopian study cohorts was how the vaccine was less immunogenic after the first vaccine dose, which suggests that there may be important differences in the response and that a two-dose regimen may be important in a non-historically endemic population.

A strong rise in GMFr as well as high seroconversion rates were noted in this study against both Inaba and Ogawa O1 strains. The vaccine is a bivalent formulation containing O1 and O139 strains. Though O139 emerged in the Bay of Bengal in 1992, it has been confined to southeast Asia. A robust response against O1 is of particular interest as it comprises the major cause of major outbreaks. However, while type-specific immunity is induced by infection, cross protective immunity between the two serotypes is incomplete.^26^

Prolonged and frequent outbreaks, increased antibiotic resistance, and raised awareness of the role of climate change in disease burden have returned cholera as a focal discussion point by the international public health community. Though improved water quality, sanitation, and hygiene measure remain the foundation of cholera prevention efforts, major improvements to infrastructure continue to be a goal far out of reach for many of those affected. Public immunization campaigns with the modified, killed, whole-cell OCV have successfully been carried out in high-risk areas across Asia, Africa, and Hispaniola.^9^,^11^,^27^,^28^ Though most trials have been conducted in Asia, this trial demonstrates that this vaccine can produce a robust immune response in less endemic populations. These trials can help pave the way for the possible use of this vaccine in any endemic region or outbreak setting throughout resource-scarce areas. Use of a cholera vaccine stockpile following disasters may offer an attractive mechanism for OCV introduction, with the hopes of yielding both public health and humanitarian benefits.^29^ As with any programmatic strategy, an effective surveillance program is paramount to assess the burden and impact of any potential outbreak. With proper disease detection programs in place, this evidence along with epidemiological data could further support the potential use of this vaccine as part of a reactive strategy in an epidemic situation. A comprehensive cholera response that links prevention with care will require a concerted international effort to reach those who are most affected and in greatest need.

## Figures and Tables

**Table 1:** Demographic characteristics

Characteristics		Adults			Children		
		Vaccine (*N* = 54)	Placebo (*N* = 54)	*P* value	Vaccine (*N* = 54)	Placebo (*N* = 54)	*P* value
Gender	Male (%)	32 (59.3)	21 (38.9)	0.03	21 (38.9)	27 (50.0)	0.25
	Female (%)	22 (40.7)	33 (61.1)	–	33 (61.1)	27 (50.0)	–
Age (years)	Mean (SD)	35.33 (8.35)	35.58 (9.72)	0.89	9.09 (4.58)	8.79 (4.52)	0.73
	Median	33.25	33.92	0.94	8.57	7.68	0.64
Weight (kg)	Mean (SD)	62.35 (12.95)	59.72 (11.79)	0.27	25.83 (12.30)	25.93 (13.13)	0.97
	Median	63.00	59.50	0.22	21.50	20.50	0.88

SD = standard deviation.

**Table 2:** Solicited systematic AEs among Ethiopian adults and children

	Vaccine	Placebo	*P* value
Adults			
Number of AEs within 3 days after first vaccine dose	0	1*	–
Number of AEs within 3 days after second vaccine dose	0	0	–
Number (%) of participants with ≥ 1 AEs 3 days following dosing regimen	0/54 (0)	1/54 (1.9)	–
Number (%) of participants with SAEs 28 days following dosing regimen	0	0	–
Children			
Number of AEs within 3 days after first vaccine dose	0	0	–
Number of AEs within 3 days after second vaccine dose	0	0	–
Number (%) of participants with ≥ 1 AEs 3 days following dosing regimen	0/54 (0)	0/54 (0)	–
Number (%) of participants with SAEs 28 days following dosing regimen	0/54 (0)	0/54 (0)	–

AEs = adverse events; SAEs = serious adverse events.

* Mild abdominal pain (*N* = 1).

**Table 3:** Vibriocidal antibody titers and proportion of ≥ 4-fold rise from baseline GMT to *V. cholerae* (include O1 Inaba, O1 Ogawa, and O139) in adults and children

Adults (aged 18 years and above)		O1 Inaba			O1 Ogawa			O139		
		Vaccine group (*N* = 54)	Placebo group (*N* = 54)	*P* value	Vaccine group (*N* = 37)	Placebo group (*N* = 46)	*P* value	Vaccine group (*N* = 46)	Placebo group (*N* = 50)	*P* value
Baseline	GMT	16.1	16.1	1	23.7	43.1	0.26	4.8	4.1	0.71
14 days after first vaccine dose	GMT	188.1	17.1	< 0.001	318.1	59.2	< 0.001	14.6	4.3	0.01
	GMF* rise	11.2	1.1	< 0.001	13.4	1.4	< 0.001	3.1	1.1	< 0.001
	No. seroconverted† (%)	37 (70)	2 (4)	< 0.001	24 (65)	5 (11)	< 0.001	13 (28)	2 (4)	0.001
14 days after second vaccine dose	GMT	254.1	18.8	< 0.001	306.4	61	< 0.001	15.1	4.2	< 0.001
	GMF* rise	15.1	1.2	< 0.001	12.9	1.4	< 0.001	3.2	1	< 0.001
No. seroconverted† (%)	43 (81)	4 (7)	< 0.001	26 (70)	6 (13)	< 0.001	14 (30)	2 (4)	0.001
Children (aged 1–17 years)		O1 Inaba			O1 Ogawa			O139		
		Vaccine group (*N* = 53)	Placebo group (*N* = 54)	*P* value	Vaccine group (*N* = 45)	Placebo group (*N* = 54)	*P* value	Vaccine group (*N* = 49)	Placebo group (*N* = 53)	*P* value
Baseline	GMT	6.2	8.5	0.48	4.2	7.2	0.17	1.6	1.6	0.92
14 days after first vaccine dose	GMT	136.8	10.8	< 0.001	143.6	7.5	< 0.001	17.1	1.6	< 0.001
	GMF* rise	21.9	1.3	< 0.001	34.6	1.1	< 0.001	10.7	1.0	< 0.001
	No. seroconverted† (%)	39 (74)	4 (7)	< 0.001	36 (80)	4 (7)	< 0.001	26 (53)	1 (2)	< 0.001
14 days after second vaccine dose	GMT	106.7	12.4	< 0.001	143.6	7	< 0.001	9.6	1.8	< 0.001
	GMF* rise	17.1	1.5	< 0.001	34.6	1	< 0.001	6	1.1	< 0.001
	No. seroconverted† (%)	41 (77)	6 (11)	< 0.001	38 (84)	3 (5)	< 0.001	21 (43)	3 (6)	< 0.001

GMT = geometric mean titers.

* Geometric mean fold rise (GMFr) from baseline to 14 days after first dose or from baseline to 14 days after second dose. Adjusted GMFr values after first or second dose to correct for difference in male:female enrolled adult vaccine and placebo recipients all remained statistically significant when compared with placebo (*P* < 0.001): O1 Inaba (11.8 and 14.5, respectively), O1 Ogawa (8.2 and 7.6, respectively), and O139 (2.9 and 3.2, respectively).

† Number with ≥ 4-fold rise in titers from baseline to 14 days after first dose or from baseline to 14 days after second dose. Primary endpoint (O1 Inaba after second dose), proportion difference between vaccine and placebo group (lower boundary of one-tailed 97.5% confidence intervals [CI]) was 74% (56%) and 66% (48%) among adults and children, respectively. Vaccine group is superior to the placebo group as the lower limit of the proportion difference is greater than clinical margin (30%).

**Table 4:** Vibriocidal antibody titers and proportion of ≥ 4-fold rise from baseline GMT to *V. cholerae* (include O1 Inaba, O1 Ogawa, and O139) in children

Children (aged 1–5 years)		O1 Inaba			O1 Ogawa			O139		
		Vaccine group (*N* = 17)	Placebo group (*N* = 17)	*P* value	Vaccine group (*N* = 16)	Placebo group (*N* = 17)	*P* value	Vaccine group (*N* = 16)	Placebo group (*N* = 17)	*P* value
Baseline	GMT	3.5	5	0.63	3.4	4.3	0.71	1.25	1.25	–
14 days after first vaccine dose	GMT	25.5	5.8	0.06	91.1	4.3	< 0.001	10	1.25	< 0.001
	GMF* rise	7.4	1.2	< 0.001	26.9	1.0	< 0.001	8	1.00	< 0.001
	No. seroconverted† (%)	11 (65)	1 (6)	< 0.001	12 (75)	2 (11)	< 0.001	7 (44)	0	0.002
14 days after second vaccine dose	GMT	15	7.4	0.38	73.4	4.1	< 0.001	6.4	1.25	0.01
	GMF* rise	4.3	1.5	0.07	21.7	1	< 0.001	5.1	1	0.01
	No. seroconverted† (%)	9 (53)	2 (11)	0.008	12 (75)	1 (6)	< 0.001	6 (37)	–	0.006
Children (aged 6–17 years)		O1 Inaba			O1 Ogawa			O139		
		Vaccine group (*N* = 36)	Placebo group (*N* = 36)	*P* value	Vaccine group (*N* = 29)	Placebo group (*N* = 36)	*P* value	Vaccine group (*N* = 33)	Placebo group (*N* = 35)	*P* value
Baseline	GMT	8.3	11	0.58	4.7	9.3	0.18	1.8	1.8	0.94
14 days after first vaccine dose	GMT	302	14.7	< 0.001	184.7	10	< 0.001	22.2	1.8	< 0.001
	GMF* rise	36.6	1.3	< 0.001	39.7	1.1	< 0.001	12.2	1	< 0.001
	No. seroconverted† (%)	28 (78)	3 (8)	< 0.001	24 (83)	2 (6)	< 0.001	19 (58)	1 (3)	< 0.001
14 days after second vaccine dose	GMT	269.1	16.2	< 0.001	208.1	9.1	< 0.001	11.8	2.1	< 0.001
	GMF* rise	32.6	1.5	< 0.001	44.7	1	< 0.001	6.5	1.2	< 0.001
	No. seroconverted† (%)	32 (89)	4 (11)	< 0.001	26 (90)	2 (6)	< 0.001	15 (45)	3 (9)	< 0.001

GMT = geometric mean titers.

* Geometric mean fold rise from baseline to 14 days after first dose or from baseline to 14 days after second dose.

† Number with ≥ 4-fold rise in titers from baseline to 14 days after first dose or from baseline to 14 days after second dose.
